# Management of External Root Resorption following Replantation After Accidental Tooth Extraction Using Regenerative Endodontic Procedures: A Case Report

**DOI:** 10.4317/jced.62832

**Published:** 2025-07-01

**Authors:** Hong Van Le, Thai Son Vu, Ngoc Linh Chi Nguyen, Kim Nha Le

**Affiliations:** 1 Department of High Technology Dental Treatment, National Hospital of Odonto-Stomatology, Hanoi, Vietnam; 2 Endodontic Department, Dental Faculty, Hanoi University of Business and Technology, Hanoi, Vietnam; 3 Department of Prosthodontic, Faculty of Dentistry, University of Medicine and Pharmacy, Vietnam National University, Hanoi, Vietnam; 4 Center for Craniofacial and Plastic Surgery, 108 Military Central Hospital , Hanoi, Vietnam

## Abstract

Accidental extraction of the wrong tooth can lead to complications such as external root resorption (ERR) after replantation. This case report describes the management of an 11-year-old female patient with ERR in a replanted mandibular first molar (#36) following iatrogenic extraction. Regenerative endodontic procedures (REPs) were employed, including chemical disinfection, intracanal calcium hydroxide medication, blood clot induction, and the placement of Biodentine™. Follow-up over 24 months showed resolution of clinical symptoms, arrest of root resorption, healing of periapical lesions, and complete apical closure. Cold pulp testing yielded positive results after 12 months, confirming regenerative response. This case highlights the potential of REPs as a biologically based approach for managing complex endodontic conditions involving root resorption, even in mature permanent teeth. It contributes to the growing body of evidence supporting REPs as a viable option in the treatment of tooth avulsion.

** Key words:**External Root Resorption, Regenerative Endodontic Procedures, Accidental Tooth Extraction.

## Introduction

Accidental extraction of the wrong tooth or teeth is considered a serious and prevenTable clinical error (1). Studies report that accidental tooth extraction accounts for approximately 20-25% of all wrong-site operations (2). Numerous causes have been reported, including miscommunication, inadequate referral, exhaustion of overworked dentists, lack of focus, and lack of experience (3,4). A mistaken extraction of a tooth should be seen as an emergency situation and managed as a case of iatrogenic traumatic avulsion. According to the International Association of Dental Traumatology (IADT) guidelines in 2020, the choice of treatment depends on factors such as the root maturity, the condition of the ligament cells which is influenced by the extra-oral time and storage condition. For an immature permanent tooth, revascularization after replantation may be considered, and endodontic treatment should be delayed. In contrast, reimplanted mature teeth should undergo endodontic treatment within 2-3 weeks after trauma to prevent potential contamination of the necrotic pulp.(5) The most common complication reported for replanted avulsed teeth is root resorption, which includes external inflammatory replacement resorption and external replacement resorption, with the former being more severe and difficult to treat (6,7). A comprehensive meta-analysis examining avulsed teeth revealed significant rates of external inflammatory root resorption (EIRR) and external replacement root resorption (ERRR), with incidences of 23.2% and 51.0%, respectively (8). EIRR manifests as a consequence of cementum and external root surface damage coupled with pulpal necrosis and infection. Timely and appropriate endodontic intervention eradicating intracanal infection can effectively halt and potentially reverse EIRR progression. The etiology of ERRR, while less definitively understood, appears to be associated with extensive periodontal attachment damage, particularly when the affected area exceeds 20% of the root surface. This underscores the importance of prompt and meticulous management of avulsed teeth to reduce the risk of resorption and improve long-term prognosis (9).

Recent findings in the treatment of EIRR have revealed the limitations of traditional methods, which have often proven unpredicTable and ineffective. Calcium hydroxide intracanal dressings, commonly used for EIRR management, can slow down the progression of resorption but fail to completely halt it. Recent studies have demonstrated that regenerative endodontic procedures (REPs) can successfully arrest both inflammatory and replacement root resorption, avoid the ankylosis complications, obtain the biologic repair particularly in cases of traumatized teeth (10-12). However, the efficacy of REPs in arresting ERRR remains uncertain, given that ERRR is primarily associated with external root damage. Some researchers have proposed that introducing vital tissue into the canal could potentially prevent progressive ERRR by providing an immune defense mechanism for the tooth (13).

This case report aims to explore the application of REPs in managing external root resorption following accidental tooth extraction and replantation, contributing to the solidity of evidence supporting this innovative approach in endodontic therapy.

Case Report

**Figure 1:**
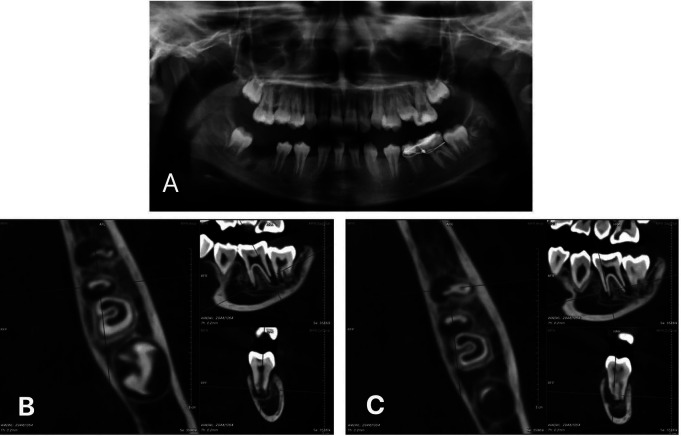
(a) Preoperative panoramic radiograph of tooth #36. (b) Preoperative CBCT scan with the slice through the distal root. (c) Preoperative CBCT scan with the slice through the mesial root.

An 11-year-old female patient was referred to the National Hospital of Odonto-Stomatology (NHOS), Hanoi from a secondary care facility. The medical history was unremarkable. The dental records indicated that the patient was initially diagnosed with a periapical cyst of tooth #46 and recommended for extraction and enucleation of the cyst under general anesthesia. However, during the operation, there was an accidental extraction of tooth #36, which was subsequently reimplanted approximately 30 minutes later and stabilized with a stainless steel wire. Two weeks post-surgery, the patient was referred to NHOS. Clinical examination revealed that tooth #36 was asymptomatic, nonresponsive to palpation and percussion, negative on vitality tests (cold and electric pulp test), and attached to tooth #35 by the stainless steel wire. Panoramic radiographs revealed no lesions related to tooth #36 (Fig. 1a). Diagnosis of pulp necrosis with a normal apical area and a history of accidental avulsion was made. The wire was removed, and the tooth was non-mobile.

The patient was scheduled for root canal treatment but she missed the appointment. Two months later, she returned to the hospital with a significant swelling on the left mandiular region. Tooth #36 was painful and mobile (grade 3), sensitive to palpation and percussion. A cone-beam computed tomography (CBCT) scan revealed radiolucency around the periapical regions of both mesial and distal roots, along with apical external root resorption at the apical third (Fig. 1b,c), leading to the diagnosis of pulp necrosis, acute apical periodontitis, and external root resorption of tooth #36.

**Figure 2:**
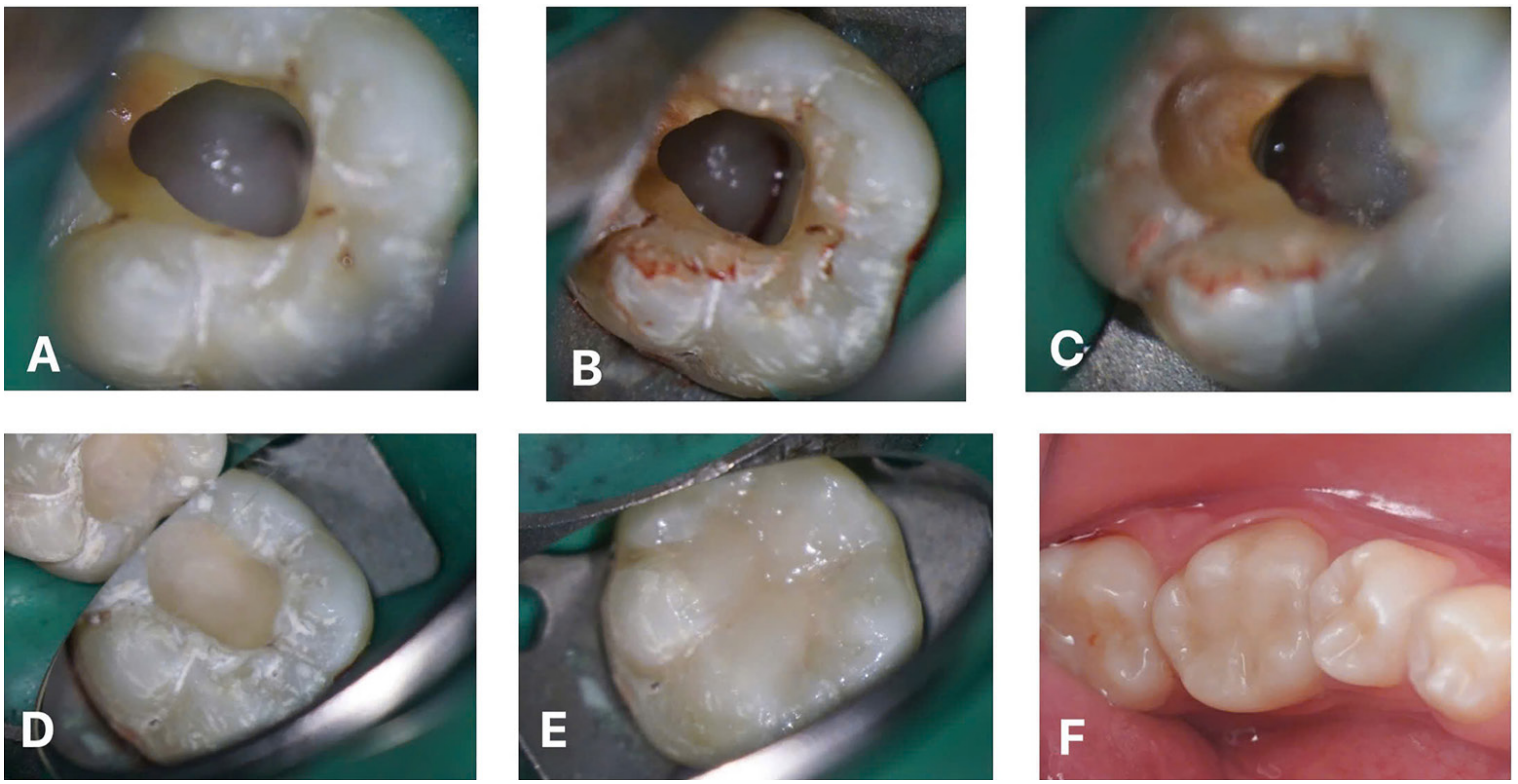
(a) Access cavity preparation during the second intervention. (b) Blood clot formation in the mesial canal under the microscope. (c) Blood clot formation in the distal canal under the microscope. (d) Placement of Biodentine™ and glass ionomer cement over the blood clot. (e,f) Final composite restoration.

**Figure 3:**
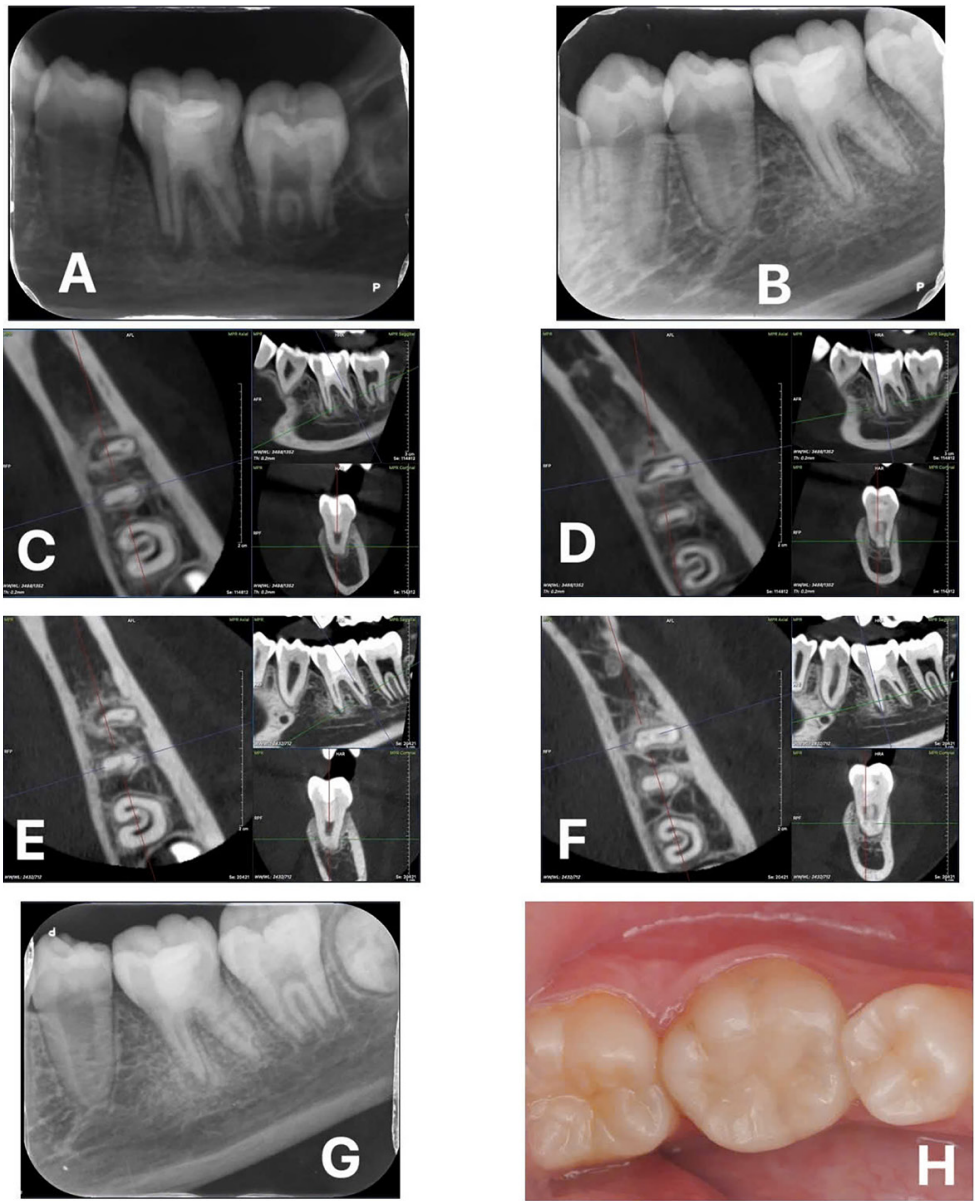
(a) Postoperative intraoral periapical radiograph. (b) 6-month follow-up intraoral periapical radiograph. (c) 12-month follow-up CBCT scan with the slice through the distal root. (d) 12-month follow-up CBCT scan with the slice through the mesial root. (e) 24-month follow-up CBCT scan with the slice through the distal root. (f) 24-month follow-up CBCT scan with the slice through the mesial root. (g) 24-month follow-up intraoral periapical radiograph. (h) 24-month follow-up clinical image showing stable composite restoration and no tooth discoloration.

Following the guidelines of the European Society of Endodontology (ESE) (14), a treatment plan involving Regenerative Endodontic Procedures (REPs) using BiodentineTM (Septodont, Saint-Maur-des-Fossés, France) was recommended over two appointments. During the first appoitment, tooth #36 was accessed after rubber dam isolation. There were no signs of vital tissues. Two mesial canals and one distal canal were identified. The working length was determined using a K file No. 10 using Intra-Oral Periapical Radiography (IOPR). The canals were irrigated with 1.5% NaOCl, activated with Ultrasonic device (IrrisafeTM tip IIRR25- Acteon Satelec). The canals were then dried with paper points and medicated with calcium hydroxide (UltraCal, Ultradent Inc., Utah, USA) for three weeks. A temporary filling with Fuji VII (GC, Japan) was placed. The second treatment appointment took place three weeks later. The tooth was asymptomatic and the temporary seal of Fuji VII remained intact. Local anesthesia without a vasoconstrictor was administered with Scandonest 3% (Septodont, Saint-Maur-des-Fossés, France), followed by rubber dam isolation. The root canals were cleaned with NaOCl 1.5% in order to remove all calcium hydroxide, followed by 20ml EDTA 17% (Coltene) for each canal. The canals were dried using sterile paper point. A sterile K file No.25 with a 3mm bent tip was employed to induce bleeding and 10-minute clot formation period was observed under the microscope (Fig. 2a,b,c). Biodentine was placed over the blood clot with a 3-mm-thick layer from the pulp floor then covered with Fuji IX (Fig. 2d) before restoring the tooth with direct composite (Fig. 2e,f). The IOPR was taken to evaluate the post-operative result (Fig. 3a). The patient underwent clinical re-examination, dental pulp testing, and IOPR imaging after 6 months (Fig. 3b), CBCT scan after 12 months (Fig. 3c,d) and 24 months (Fig. 3e,f) post-treatment. The follow-up examinations indicated the absence of clinical symptoms, with painless response from percussion and palpation tests on tooth #36. Positive responses were observed in cold pulp tests after 12 months, and these results remained sTable at the 24-month follow-up. The IOPR showed a normal periapical appearance (Fig. 3g). There were no color changes noted in the tooth, and the composite filling appeared stable (Fig. 3h).

The CBCT taken after 12 months revealed that the external resorption had ceased, with complete healing of the periapical lesion. Subsequent CBCT images at 24 months showed consistent and stable outcomes of periapical lesion, the dentine was regenerated right at the root resorption area, the apices were completely closed.

## Discussion

Accidental tooth extraction during surgical procedures may result in severe complications, including the inadvertent extraction of a healthy tooth, as occurred in this case where tooth #36 was mistakenly extracted instead of tooth #46. This underscores the critical role of thorough pre-operative planning, proper communication within the surgical team, and strict adherence to protocols for tooth identification and verification to prevent such errors (1). Studies have documented similar incidents of wrong tooth extraction, emphasizing the need for stringent compliance with established guidelines to minimize medical errors (15).

The external root resorption (ERR) observed in this case is a common complication following tooth avulsion and replantation (16). Abbott *et al*. (2022) described ERR as the destruction of dentin and cementum, often accompanied by bone loss. The “Resorption Triad,” which comprises the breakdown of natural tissue barriers, the presence of a continuous stimulating factor, and a viable blood supply for clastic cells, is essential for the resorption process to occur (17). In this patient, the pathophysiology of ERR was initiated by trauma to the periodontal ligament (PDL) during the accidental extraction. Damage to the PDL exposed the root surface and dentinal tubules, triggering the activation of osteoclasts and odontoclasts (18). The tooth was replanted approximately 30 minutes after avulsion, while relatively short, is suboptimal if the PDL cells had not been preserved properly. The absence of documentation on the storage conditions of the tooth further complicates the assessment. A suboptimal reimplantation condition is likely to contribute to an inflammatory response and subsequent ERR. The diagnosis of pulp necrosis and postponed endodontic treatment allowed bacterial toxins to infiltrate the periapical tissues, stimulating inflammatory cytokines (e.g., TNF-α, IL-1, IL-6) and activating the RANKL pathway, which promotes osteoclastic activity and root resorption (19,20).

The decision to perform REPs using BiodentineTM in this case was based on the clinical presentation of pulp necrosis, acute apical periodontitis, and ERR (5). While REPs are not primarily indicated for ERR, they have been shown to arrest resorption and promote periapical healing by creating a biologically active environment within the root canal system (13,21,22). The elimination of necrotic tissue using 1.5% NaOCl with ultrasonic activation, followed by intracanal calcium hydroxide medication, created favorable conditions for healing. According to the European Society of Endodontology (ESE) guidelines, chemical disinfection without mechanical instrumentation is critical in REPs to preserve the fragile root structure and apical papilla (23). The second appointment involved the use of 17% EDTA as the final irrigant, which has been shown to enhance the survival and differentiation of stem cells from the apical papilla (SCAPs). Meeprasert *et al*. (2023) demonstrated that EDTA mitigates the harmful effects of NaOCl and enhances SCAPs viability, supporting tissue regeneration (24,25).

The successful outcome in this case, including the cessation of ERR, resolution of the periapical lesion, and regeneration of functional pulp tissue, can be attributed to the comprehensive treatment approach. The systematic follow-up examinations at 6, 12, and 24 months confirmed the resolution of clinical symptoms, positive pulp vitality responses, and radiographic evidence of apical closure and dentin regeneration. Numerous studies by independent research groups such as Scelza *et al*. (2021), Chrepa *et al*. (2020) and Theekakul C. *et al*. (2024) reported similar outcomes, with positive cold test responses in 41-68% of cases following REPs (25-27).

The regenerative process in this 11-year-old patient relied on the survival of Hertwig’s epithelial root sheath and the differentiation of SCAPs into odontoblast-like cells under the stimulation of Biodentine™. These cells contributed to the formation of dentin-like tissue at the resorption site, promoting apex closure and strengthening the tooth structure. The interaction between bioactive materials and growth factors during REPs further enhanced the recruitment and differentiation of stem cells, leading to the resolution of periradicular lesions and restoration of the tooth’s structural integrity (28-31). Patients under 12 years old were a significant prognostic factor for healing, as reported in Mahidol study (25).

The European Society of Endodontology (ESE) and the American Association of Endodontists (AAE) provide guidelines for evaluating the success of REPs, emphasizing a comprehensive assessment approach. Key criteria include the resolution of clinical symptoms, radiographic evidence of periapical healing and lesion reduction, positive pulp vitality testing, maintenance of tooth integrity and stability, and preservation of periodontal health (32,33). In this 24- month follow-up case, all AAE and ESE clinical and radiographic criteria were met, including the evidence of vitality of the newly regenerated pulp tissue and the absence of tooth discoloration. This favorable outcome can be attributed to the use of BiodentineTM, which has been documented in numerous studies to minimize discoloration while supporting tissue regeneration (34,35).

## Conclusions

This case highlights the effectiveness of REPs in the management of complex conditions involving pulp necrosis, apical periodontitis, and ERR. The biologically based approach not only preserved the natural tooth but also achieved functional and structural regeneration, fulfilling the clinical and radiographic success criteria outlined by the ESE and AAE. The use of Biodentine™ and adherence to established protocols played a pivotal role in the favorable outcomes observed in this 24-month follow-up case.

## Data Availability

The datasets used and/or analyzed during the current study are available from the corresponding author.
